# NLR Risk Score for Predicting Patient Prognosis in Hepatocellular Carcinoma and Identification of Oncogenic Role of NLRP5 in Hepatocellular Carcinoma

**DOI:** 10.32604/or.2025.067065

**Published:** 2025-09-26

**Authors:** Mingyang Tang, Shengfu He, Bao Meng, Qingyue Zhang, Chengcheng Li, Yating Sun, Weijie Sun, Cui Wang, Qingxiang Kong, Yanyan Liu, Lifen Hu, Yufeng Gao, Qinxiu Xie, Jiabin Li, Ting Wu

**Affiliations:** 1 Department of Infectious Diseases, The First Affiliated Hospital of Anhui Medical University, Hefei, 230022, China; 2 Anhui Province Key Laboratory of Infectious Diseases, Anhui Medical University, Hefei, 230022, China; 3 Department of Gastroenterology, The First Affiliated Hospital of Anhui Medical University, Hefei, 230022, China; 4 Department of Critical Care Medicine, The First Affiliated Hospital of Anhui Medical University, Hefei, 230022, China; 5 Department of Infectious Diseases, Chaohu Hospital of Anhui Medical University, Hefei, 230022, China

**Keywords:** Hepatocellular carcinoma, nod-like receptor family, NLR family pyrin domain containing 5, Tumor protein 53

## Abstract

**Background:**

Hepatocellular carcinoma (HCC) is a major cause of cancer-related deaths. The Nod-like receptor (NLR) family is involved in innate immunity and tumor progression, but its role in HCC remains unclear. This study aimed to evaluate the prognostic value and biological function of NLR genes in HCC.

**Methods:**

Transcriptomic and clinical data from The Cancer Genome Atlas were analyzed using nonnegative matrix factorization (NMF) to classify HCC into molecular subtypes. Differentially expressed genes were used to build an NLR-based prognostic model (NLR_score) through univariate Cox, least absolute shrinkage and selection operator (LASSO), and multivariate Cox regression. Predictive performance and correlation with chemotherapy sensitivity were assessed. NLR family pyrin domain containing 5 (NLRP5) was identified as a key oncogene and validated via *in vitro* assays, including cell counting kit-8 (CCK-8), colony formation, transwell, and flow cytometry *in vivo* xenograft models.

**Results:**

The two NMF-defined subtypes showed distinct survival outcomes. The NLR_score reliably predicted prognosis and was associated with sensitivity to six chemotherapeutic drugs. NLRP5 knockdown suppressed HCC cell proliferation, migration, and invasion *in vitro* and reduced tumor growth *in vivo*. Mechanistically, NLRP5 modulated the p53 signaling pathway, influencing cell cycle and apoptosis.

**Conclusion:**

This study developed an NLR-based prognostic model that effectively stratifies HCC patients by survival risk. NLRP5 was identified as a novel oncogene promoting HCC progression via the p53 pathway, suggesting its potential as a therapeutic target.

## 1 Introduction

Hepatocellular carcinoma (HCC) is the predominant subtype of hepatic malignancy and one of the most common malignant tumors worldwide, with a high mortality rate [1]. Despite significant advances in HCC research, its insidious onset and lack of specific early symptoms often result in diagnosis at advanced stages or after distant metastasis has occurred. As a result, the clinical efficacy and prognosis of HCC are still poor [2–4]. Currently, the three-year survival rate for HCC patients is only 50% [5]. Hence, it is urgent to explore and develop innovative biomarkers for diagnosis, prognosis, and treatment to ameliorate the medical outcomes of individuals with HCC.

The Nod-like receptor (NLR) family is a pattern recognition receptor located in the cytoplasm. The composition consists of three types of domains with different functions: a leucine-rich repeat (LRR) domain located at the C-terminus, a nucleotide-binding and oligomerization domain (NACHT) positioned in the middle, and an N-terminal effector domain [6,7]. The NLR family can be categorized into NLRA, NLRB, NLRC, NLRP, and NLRX subfamilies based on their distinct N-terminal domains. Notably, the NLRP subfamily plays a role in the assembly of inflammasomes [8]. The inflammasome usually converts immature pro-interleukin-18 (IL-18) and IL-1β into biologically active IL-18 and IL-1β by activating caspase-1. Moreover, the inflammasome also regulates caspase-1/gasdermin D-dependent pyroptosis [9,10]. After considerable research, the number of known members of the inflammasome family is still expanding, while enhancing our comprehension. Recent research shows that inflammasomes are closely related to inflammatory diseases, neurological diseases, and cancer [11,12]. NLR family pyrin domain containing 3 (NLRP3) has been extensively studied among them, due to its impressive functions. Furthermore, NLRP3 has garnered widespread attention in liver diseases such as liver fibrosis [13], non-alcoholic fatty liver disease [14], acute and chronic cholestatic liver injury [15], liver ischemia and reperfusion injury [16], liver regeneration after drug-induced liver injury [17] and liver cancer [18]. Considering the significance of NLRP3, it is imperative to perform a systematic and comprehensive study of the NLR family gene in HCC.

This study aimed to comprehensively investigate the role of NLR family genes in HCC. Specifically, we sought to classify distinct NLR-based molecular subtypes and explore their association with patient prognosis and the tumor immune microenvironment. We also aimed to construct a prognostic scoring system (NLR_score) for risk stratification and treatment guidance. Furthermore, we investigated the functional role of NLR family pyrin domain containing 5 (NLRP5) in HCC progression. This study provides a basis for understanding the clinical relevance of NLRs and evaluating NLRP5 as a potential therapeutic target in HCC.

## 2 Materials and Methods

### 2.1 Data Download and Processing

Gene expression profiles and clinical prognosis data of HCC patients were obtained from The Cancer Genome Atlas (TCGA, https://portal.gdc.cancer.gov/ (accessed on 29 June 2025)) and the International Cancer Genome Consortium (ICGC, https://dcc.icgc.org/ (accessed on 29 June 2025)) databases. Somatic mutation and copy number variation (CNV) data were acquired from the TCGA-HCC cohort. Correlation analysis was conducted after removing patients with incomplete clinical information. Furthermore, patients with an overall survival (OS) of less than 30 days were excluded from the prognostic analysis in order to avoid bias caused by non-tumor-related mortality, such as perioperative complications or unrelated acute conditions. In addition, single-cell RNA sequencing (scRNA-seq) data of HCC were retrieved from the Gene Expression Omnibus (GEO, https://www.ncbi.nlm.nih.gov/gds/ (accessed on 29 June 2025)) database under accession number GSE146115.

### 2.2 Graphics Drawing

In this study, the ‘ggplot2’ (version 3.5.2) package was used to draw the volcano map, bubble chart, and violin plots ‘pheatmap’ (version 1.0.12) package was used to draw the heat map. The ‘RCircos’ (version 1.2.1) package was used to draw the circular diagram. The ‘Seurat’ (version 5.3.0) package to draw Feature plots.

### 2.3 The Determination of the Subtypes of Nonnegative Matrix Factorization

It is a powerful method for classifying diseases based on the expression of specific genes in nonnegative matrix factorization (NMF) [19]. With the ‘NMF’ package (version 0.23.0), the study utilized NLR-related gene expression to identify subtypes of HCC patients, and the optimal number of clusters was finally determined to be 2 based on cophenetics. We divided HCC patients into Cluster 1 (C1) and Cluster 2 (C2) by the optimal number of clusters. Finally, we used Kaplan-Meier to draw the survival curves of C1 and C2 patients.

### 2.4 Processing of the scRNA-Seq Data

The scRNA-seq data processing was performed according to our previous treatment methods [20]. In brief, the study used the ‘Seurat’ package (version 5.3.0) to preprocess and analyze the data [21]. This study eliminated cells with fewer than 1000 genes or cells with more than 5% mitochondrial genes. Calculation of mitochondrial genes in a cell was done using the PercentageFeatureSet. The principal component was set to 20, and the resolution was set to 0.2. t-SNE was used for dimensionality reduction and visualization of the data. For cell clustering, 1500 genes with the greatest differences between cells were used. The ‘SingleR’ (version 2.4.1) package was used for automatic annotation of cell types.

### 2.5 Basic Ontology Analysis and Kyoto Encyclopedia of Genes and Genomes (KEGG) Analysis

The analysis of differentially expressed genes involved the examination of biological processes (BPs), cellular components (CCs), and molecular functions (MFs). Additionally, the KEGG analysis and Gene Set Variation Analysis (GSVA) analysis were conducted to explore the signal transduction pathways of the differentially expressed genes. The Gene Ontology (GO) and KEGG analyses were performed by utilizing the packages ‘clusterProfiler’ (version 4.10.1), ‘org. Hs. eg. db.’ (version 3.18.0), ‘enrichplot’ (version 1.22.0), ‘GSVA’ (version 1.50.5), and ‘GSEABase’ (version 1.64.0).

### 2.6 Construction and Validation of NLR Score

Based on the ‘survival’ software package (version 3.8.3), the differentially expressed genes (DEGs) between C1 and C2 samples were filtered and screened by univariate Cox analysis, least absolute shrinkage and selection operator (LASSO) analysis, and multivariate Cox analysis. In LASSO regression, we use the k-fold cross-validation (10-fold cross-validation) method to select the regularization parameter λ, which is implemented by the R package ‘glmnet’ (version 4.1.8). At the same time, based on the results of multivariate Cox analysis, we established a five-gene optimal prognostic model (NLR score). The calculation formula of NLR score for each HCC patient is as follows:

$$NLR\;score=\sum_{n=1}^{N}\text{Expression}\left(\text{i}\right)*Coefficient\;(i)$$

where the coefficient is the importance score of the selected gene in the results of multivariate Cox regression analysis.

### 2.7 Somatic Mutation Spectrum Analysis

This study employed the ‘maftools’ package (version 2.24.0) to calculate somatic mutations in the TCGA-HCC sample data [22]. Based on the results, the study analyzed the top 10 genes most prone to mutation in patients with varying NLR subtypes and NLR_score groups, ultimately visualizing them through a waterfall chart.

### 2.8 Evaluation of Immune Cell Infiltration

The study utilized the Single Sample Gene Set Enrichment Analysis (ssGSEA) algorithm to assess the levels of infiltration of 28 tumor-infiltrating lymphocytes in the TCGA-HCC cohort. The level of immune cell infiltration was compared among different NLR subtypes and NLR_score. The Tumor Immune System Interaction Database (TISIDB, http://cis.hku.hk/TISIDB/download.php (accessed on 29 June 2025)) was applied to download gene signatures of tumor-infiltrating lymphocytes.

### 2.9 Drug Sensitivity Analysis

The ‘oncopredict’ package (version 1.2) was employed in this study to forecast the IC_50_ values of six commonly used chemotherapeutic agents for HCC, including Sorafenib, Cisplatin, Fludarabine, Oxaliplatin, Gemcitabine, and 5-Fluorouracil [23]. Subsequently, the differences in the IC_50_ values among the six drugs were assessed across various NLR_score groups.

### 2.10 Cell Culture and Transfection

The HCCLM3 and HepG2 human liver cancer cell lines used in this study were originally obtained from the Cell Bank of the Chinese Academy of Sciences (Shanghai, China). Both cell lines have been authenticated by short tandem repeat (STR) profiling and regularly tested negative for mycoplasma contamination. The cells have been maintained and cultured in our laboratory under standard conditions [20]. Complete Dulbecco’s Modified Eagle Medium (DMEM, Thermo Fisher Scientific, Cat No. 11965092, Waltham, MA, USA) supplemented with 10% fetal bovine serum (FBS, Thermo Fisher Scientific, Cat No. A5256701) and 1% penicillin-streptomycin (Beyotime, Cat No. C0222, Shanghai, China) was used for culturing all cell lines. Beijing Tsingke Biotech (Beijing, China) designed and manufactured short interfering RNAs (siRNA). Before usage, the siRNA powder was dissolved in RNase-free water. The siRNA was then diluted with Optimized Minimum Essential Medium (Opti-MEM, Thermo Fisher Scientific, Cat No. A5256701) to the working concentration (50 nM), and the Lipofectamine™ 2000 solution (Thermo Fisher Scientific, Cat No. 11668019) was prepared. Subsequently, a mixture of siRNA and Lipofectamine™ 2000 was incubated at ordinary temperature for 20 min. Simultaneously, the cell culture medium was substituted with serum-free Opti-MEM. After waiting for 6 h, the transfection medium was replaced with complete DMEM medium, and further experiments were carried out 48 h after transfection. The sequences of all siRNA employed in this investigation are presented in Table 1.

**Table 1:** siRNA sequences

Gene	Sense (5^′^-3^′^)	Antisense (5^′^-3^′^)
Si-NLRP4-1	GAUCAUUCAAGGACCACAA	UUGUGGUCCUUGAAUGAUC
Si-NLRP4-2	CAACGAGAGUGAUAGGUUA	UAACCUAUCACUCUCGUUG
Si-NLRP4-3	GAAUGUGGGUUAACGAGCA	UGCUCGUUAACCCACAUUC
Si-NLRP5-1	GGCUUCCGAUUAACCAGAA	UUCUGGUUAAUCGGAAGCC
Si-NLRP5-2	GCAUGAGUAUUAUGGAGCA	UGCUCCAUAAUACUCAUGC
Si-NLRP5-3	GAAGGAGACAAAUCGCUCA	UGAGCGAUUUGUCUCCUUC
Negative control	UGGUUUACAUGUCGACUAA	UUAGUCGACAUGUAAACCA

### 2.11 RNA Extraction, Reverse Transcription, Quantitative Reverse Transcription Polymerase Chain Reaction (qRT-PCR), and RNA Sequencing

TRIzol reagent was utilized to extract the total RNA from the HCCLM3 cell lines with/without siRNA in accordance with the guidelines provided by the manufacturer (Thermo Fisher Scientific, Cat No. 15596026). The RNA isolation and qRT-PCR protocols were performed following the previously stated methods [24]. The primer sequences employed are provided in Table 2. The RNA sequencing analysis was performed by the Beijing Genomics Institute (BGI-Shenzhen, China) utilizing the BGISEQ-500 platform, as previously reported [24].

**Table 2:** qRT-PCR primers

Gene	Forward primer (5^′^-3^′^)	Reverse primer (5^′^-3^′^)
ACTB	CACCATTGGCAATGAGCGGTTC	AGGTCTTTGCGGATGTCCACGT
NLRP4	ATCTTTCGGGATAGGTTCCTGT	ACACGATCTCTGTTATAGGAGCA
NLRP5	CTGGACGCCTTCCACTGTCTTT	CGCAAATACGGACAGTGCTGGA

### 2.12 Cell Counting Kit-8 (CCK-8) Assay

After transfection treatment, HCCLM3 and HepG2 cells were cultured in DMEM Medium supplemented with 10% FBS in a humidified incubator at 37°C with 5% CO_2_. A total of 5000 cells were seeded into each well of a 96-well plate. At 0, 24, 48, 72, and 96 h post-seeding, 10 µL of CCK-8 working solution (Beyotime, Cat No. C0037) was added to each well, followed by incubation for an additional 2 h under standard culture conditions. The absorbance was measured at 450 nm using Tecan Spark (Männedorf, Switzerland). Each group was tested in six replicate wells, and the experiment was repeated independently at least three times.

### 2.13 Colony Formation Assay

A sum of 1000 HCCLM3 and HepG2 cells was gathered subsequent to undergoing transfection treatment, subsequently amalgamated, and placed in a 6-well plate. A sufficient quantity of culture medium was added, and the cells were grown for a period of 10 days before ending the experiment. Afterward, the cells were washed with 1× PBS (pH 7.4), then treated with 4% paraformaldehyde to fix them for 15 min, and stained with 0.1% crystal violet for the same duration. Finally, images were captured to document the number of cell clusters formed using ImageJ software (Version 1.53t, National Institutes of Health, Bethesda, MD, USA).

### 2.14 Transwell Assay

During the migration experiment, a suspension of 5 × 10^4^ HCCLM3 and HepG2 cells in DMEM medium with 1% FBS was inserted into the upper layer of the transwell chamber. Simultaneously, 800 µL of culture medium with 20% FBS was poured into every well of the lower chamber within a 24-well plate. In the invasion experiment, an additional 100 µL of Matrigel (Corning, Cat No. 356234, Corning, NY, USA) was added to the top layer of the transwell chamber. After a 24-h period of incubation, the cells on the filter surface were gently removed using a cotton swab and then treated with 4% paraformaldehyde for 15 min to fix. Afterwards, the cells were stained with 0.1% crystal violet dye for a duration of 20 min, and images were captured by microscopic observation (TissueFAXS Plus S, TissueGnostics, Vienna, Austria).

### 2.15 Western Blot

The HCCLM3 cells underwent two washes with cold 1× PBS (pH 7.4) and were subsequently lysed in radio-immunoprecipitation assay lysis (RIPA) buffer (Beyotime, Cat No. P0013) supplemented with the protease inhibitor phenylmethanesulfonyl fluoride (PMSF) (Beyotime, Cat No. P0013). The bicinchoninic acid (BCA) Protein Assay Kit (Beyotime, Cat No. P0011) was utilized to determine the protein concentration in cell lysate in accordance with the instructions provided by the manufacturer. The cell lysate was mixed with SDS loading buffer and heated to 100°C for 10 min. Subsequently, the cell lysate was separated by SD-PAGE, and the proteins were transferred onto a polyvinylidene fluoride (PVDF) membrane (Merck, Cat No. ISEQ00010, Darmstadt, Germany) that had been previously treated with methanol. Afterward, the membrane was blocked using 5% skim milk for 1 h and subsequently exposed to primary antibodies at 4°C overnight. The antibodies employed were as listed: NLRP5 (1:1000; Cat No. PA5-21021, Thermo Fisher Scientific); p53 (1:500; Cat No. AF0879, Affinity, Changzhou, China); MDM2 (1:1000; Cat No. 51541, CST, Danvers, MA, USA); Bax (1:2000; Cat No. 50599, Proteintech, Rosemont, IL, USA); P21 (1:2000; Cat No. 10355, Proteintech); and β-actin (1:3000; Cat No. T0022, Affinity). The membrane was washed three times with TBST (Biosharp, Cat No. BL346A, Hefei, China) for 10 min each, then incubated with an HRP-labeled secondary antibody (1:5000; Cat No. SA00001-1/2, Proteintech) for 1 h at room temperature. The protein bands were subsequently identified using an enhanced ECL chemiluminescence (Thermo Fisher Scientific, Cat No. 34580) detection system.

### 2.16 Cell Cycle and Apoptosis Analysis

The cell cycle was evaluated utilizing a Cell Cycle and Apoptosis Analysis Kit (Beyotime, Cat No. C1052), while the cell apoptosis was measured using an Annexin V-FITC Apoptosis Detection Kit (Beyotime, Cat No. C1062) following the manufacturer’s guidelines. The HCCLM3 Cells were collected 48 h post-transfection with siRNA. In order to examine the cell cycle, cells were first exposed to cold 75% ethanol and then kept at 4°C for 24 h to ensure fixation. Afterward, every group was subjected to a prepared 1× propidium iodide solution and placed in the dark for 30 min. To investigate cell apoptosis, 1× Annexin V-FITC and propidium iodide staining solution were added, and then the cells were gently mixed. Following that left to incubate dimly at ambient temperature for 20 min. Ultimately, FACS Celesta (BD, San Jose, CA, USA) was employed to collect data, which was subsequently analyzed utilizing FlowJo software (Version 10.10, BD, Franklin Lakes, NJ, USA).

### 2.17 Animal Models

C57BL/6 mice carrying the H11-LSL-MYC and Alb-Cre transgenes were obtained from the Shanghai Model Organisms Center and maintained in the specific pathogen-free (SPF) animal facility of Anhui Medical University. By crossing these two strains, we generated Alb-Cre^+^/MYC^+^ mice, which spontaneously develop primary liver tumors, as previously described [25]. A total of 12 male Alb-Cre^+^/MYC^+^ mice (aged 6 weeks, weighing 22 ± 2 g) were randomly divided into two groups: the NLRP5-downregulated group (n = 6) and the control group (n = 6). Mice were housed in individually ventilated cages under SPF conditions, maintained at a constant temperature of 20°C–25°C and 50% ± 5% humidity, with a 12-h light/dark cycle. All animals had free access to autoclaved chow and sterile water. To knock down NLRP5, mice in the treatment group were intravenously injected with 5 nmol *in vivo*-grade cholesterol-conjugated siRNA targeting NLRP5 (Sequence: CAAUGAGUCUGAUAGAAUA, RiboBio, Guangzhou, China) twice weekly for 3 consecutive weeks. Mice were sacrificed at 9 weeks of age. Euthanasia was performed via inhalation of 20% carbon dioxide until complete loss of consciousness was observed, followed by cervical dislocation to ensure death. All animal experiments were approved by the Ethics Committee of Anhui Medical University (Approval No. LLSC20230063) and conducted in accordance with institutional and national guidelines for the care and use of laboratory animals.

### 2.18 Immunohistochemical Staining

Mouse liver cancer tissues were washed with 1× PBS (pH 7.4), then fixed in 4% paraformaldehyde overnight, dehydrated, and embedded. The resulting samples were then sliced into sections measuring 0.4 µm in thickness and were dewaxed and then treated with 10 mM citrate antigen retrieval solution (pH 6.0) in a microwave oven for 15 min to facilitate antigen retrieval. After undergoing natural cooling, the sections were subjected to a 25-min treatment with a 3% H_2_O_2_ solution to suppress the activity of endogenous peroxidase. Afterward, the sections were left to incubate in a solution containing 3% bovine serum albumin (BSA, Beyotime, Cat No. P007) at ambient temperature for 1 h. The primary antibodies, anti-Ki67 (1:750, Cat No. GB111499-100, Servicebio, Shanghai, China) and anti-NLRP5 (1:100, Cat No. PA5-21021, Thermo Fisher Scientific), were incubated with the samples overnight at 4°C. After that, sections were rinsed with 1× PBS (pH 7.4) and then incubated with a secondary antibody (1:200; Cat No. GB23303, Servicebio) for 1 h at ordinary temperature. In the end, the samples were subjected to 0.05% diaminobenzidine (DAB) staining for visualization (Servicebio, Cat No. G1211) and followed by nuclear counterstaining using 0.1% hematoxylin (Servicebio, Cat No. G1004). The images were captured through microscopic observation (TissueFAXS Plus S, TissueGnostics, Vienna, Austria). The analysis of the image was performed by a person who was unaware of the grouping, utilizing ImageJ software. The levels of Ki67 and NLRP5 protein expression were determined by calculating the ratio of the positive area percentage.

### 2.19 Terminal Deoxynucleotidyl Transferase dUTP Nick-End Labeling (TUNEL) Assay

Apoptosis in mouse liver tissue was assessed utilizing the TUNEL Apoptosis Detection Kit (Yesen, Cat No. 40306ES20, Shanghai, China) following the manufacturer’s instructions. To remove paraffin, paraffin-embedded tissue sections underwent a step-by-step process using xylene, ethanol, and distilled water, respectively. Afterward, 20 μg/mL Proteinase K was added slowly and left to incubate at room temperature for 20 min. After the 1× PBS (pH 7.4) washing, samples were treated with 1× TdT incubation buffer and then incubated at 37°C for 60 min. Subsequently, the samples were treated with 1× equilibration buffer and incubated at room temperature for 30 min, protected from light. Finally, all samples were counterstained with 0.5 μg/mL DAPI (Beyotime, Cat No. C1006) and then sealed using an anti-fluorescence quencher (Beyotime, Cat No. P0126). Images were promptly observed and captured using Zeiss LSM980 (Carl Zeiss Microscopy GmbH, Jena, Germany).

### 2.20 Statistical Analysis

All bioinformatics analyses were conducted using R software (version 4.2.3). Spearman correlation analysis was performed to assess associations between variables. Kaplan–Meier survival analysis was used to generate survival curves. For statistical comparisons, the Wilcoxon rank-sum test was applied to non-normally distributed data, while normally distributed data were analyzed using the unpaired Student’s *t*-test. Data are presented as the Mean ± Standard Error of the Mean (SEM). Comparisons among multiple groups were performed using one-way analysis of variance (ANOVA). A *p*-value of less than 0.05 was considered statistically significant.

## 3 Results

### 3.1 Distribution of NLR Family Genes and Establishment of NLR Subtypes

**Figure 1:**
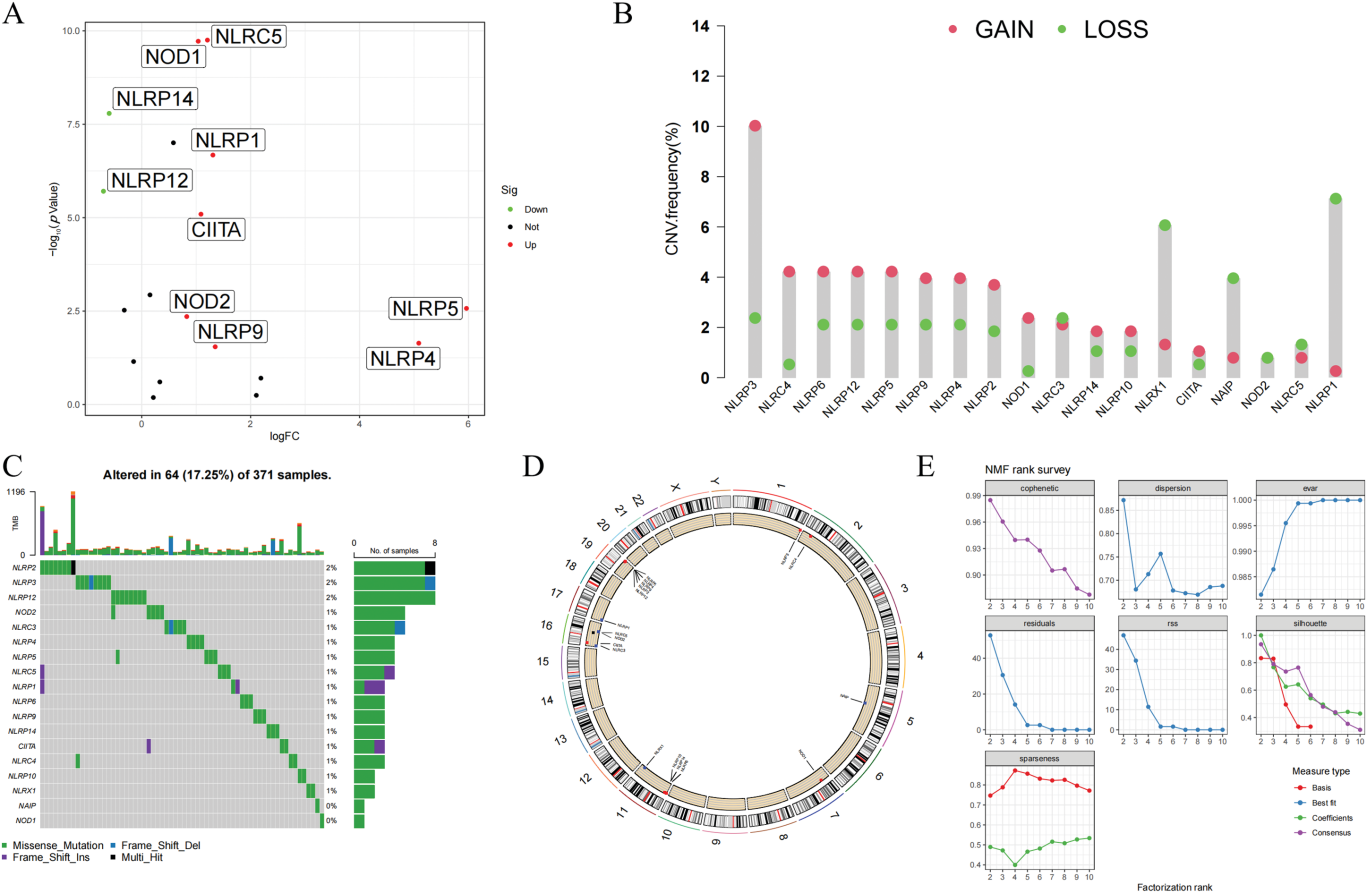

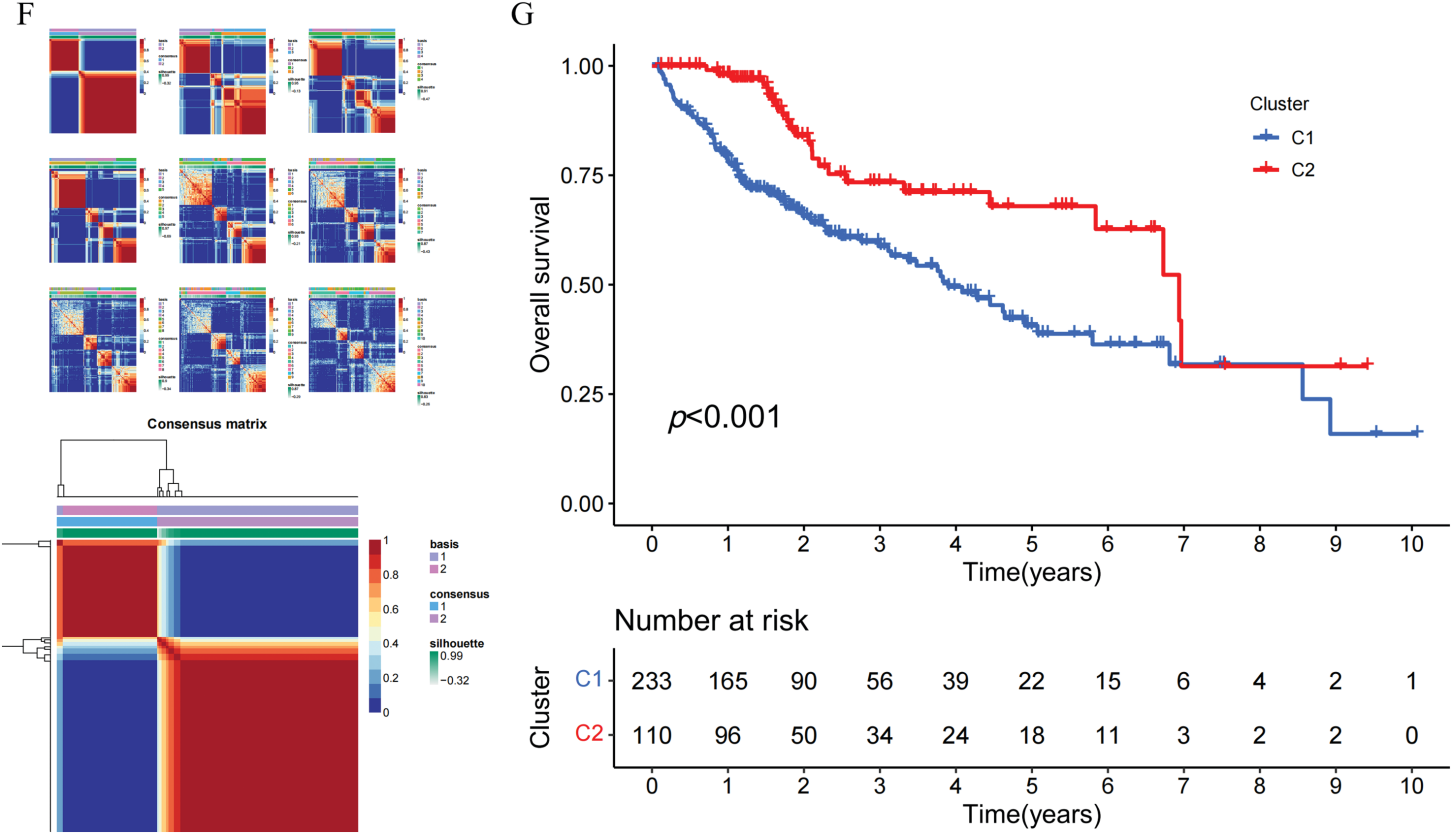
Genomic landscape and molecular classification of NLR family genes in HCC. (**A**) Differential expression analysis of NLR family genes between HCC and normal tissues. Significantly upregulated and downregulated genes are indicated. (**B**) Copy number variation (CNV) frequency of NLR genes in HCC samples, showing both gain (**red**) and loss (**green**) events. (**C**) Somatic mutation landscape of NLR family genes in HCC. The top panel shows mutation counts per sample, and the lower panel displays mutation types for each gene. (**D**) Circos plot illustrating the chromosomal locations of NLR family genes with CNV and mutation annotations. (**E**) Determination of the optimal number of clusters (k) using non-negative matrix factorization (NMF). The cophenetic coefficient supported k = 2 as the most stable clustering solution. (**F**) Consensus clustering results for k = 2 to k = 10, and the final consensus matrix heatmap for k = 2. (**G**) Kaplan-Meier survival analysis comparing overall survival (OS) between the two NLR-based molecular subtypes (C1 and C2) in the TCGA-HCC cohort. Patients in Cluster C1 exhibited significantly poorer OS than those in Cluster C2 (*p* < 0.001)

In this study, a total of 18 NLR family genes were selected for further examination [26]. These genes include NLRP1, NLRP2, NLRP3, NLRP4, NLRP5, NLRP6, NLRP9, NLRP10, NLRP12, and NLRP14 from the NLRP family; NLRA family member class II major histocompatibility complex transactivator (CIITA); NLRB family member NLR family apoptosis inhibitory protein (NAIP); nucleotide-binding oligomerization domain-containing protein 1 (NOD1), NOD2, NOD3, NOD4, and NLRC5 from the NLRC family; and NLRX family member NLRX1. The study initially visualized the expression condition of the NLR family genes using volcano plots. The results indicated that 8 NLR family members exhibited high expression in HCC, while 2 NLR family members displayed low expression in HCC (Fig. 1A). Furthermore, the study examined the incidence of CNV and somatic mutations in NLR family genes. CNVs were observed in all NLR family members, with NLRP3 exhibiting the most CNV gain and NLRP1 showing the least CNV loss (Fig. 1B). Concerning somatic mutations, the mutation frequency of NLR family members in HCC is low. NLRP2, NLRP3, and NLRP12 had the highest mutation frequencies among NLR family genes, all at 2%, with the majority of mutations being nonsense mutations (Fig. 1C). In addition, the study employed a circular diagram to visualize the CNV alteration locations of NLR family members across the chromosomes (Fig. 1D). Moreover, the TCGA-HCC cohort was divided into distinct NLR subtypes through the utilization of unsupervised consensus clustering analysis, taking into account the expression levels of NLR family members. Cophenetic selection was used to determine the optimal number of clusters, which was k = 2 (Fig. 1E). Finally, the study provided consensus matrix heatmaps for varying cluster numbers. Subsequently, the Kaplan-Meier analysis demonstrated a statistically notable decrease in the overall survival (OS) within C1 compared to C2 (Fig. 1G).

### 3.2 Distribution of the NLR Family Gene in the Transcriptional Profiles of HCC Single Cells

**Figure 2:**
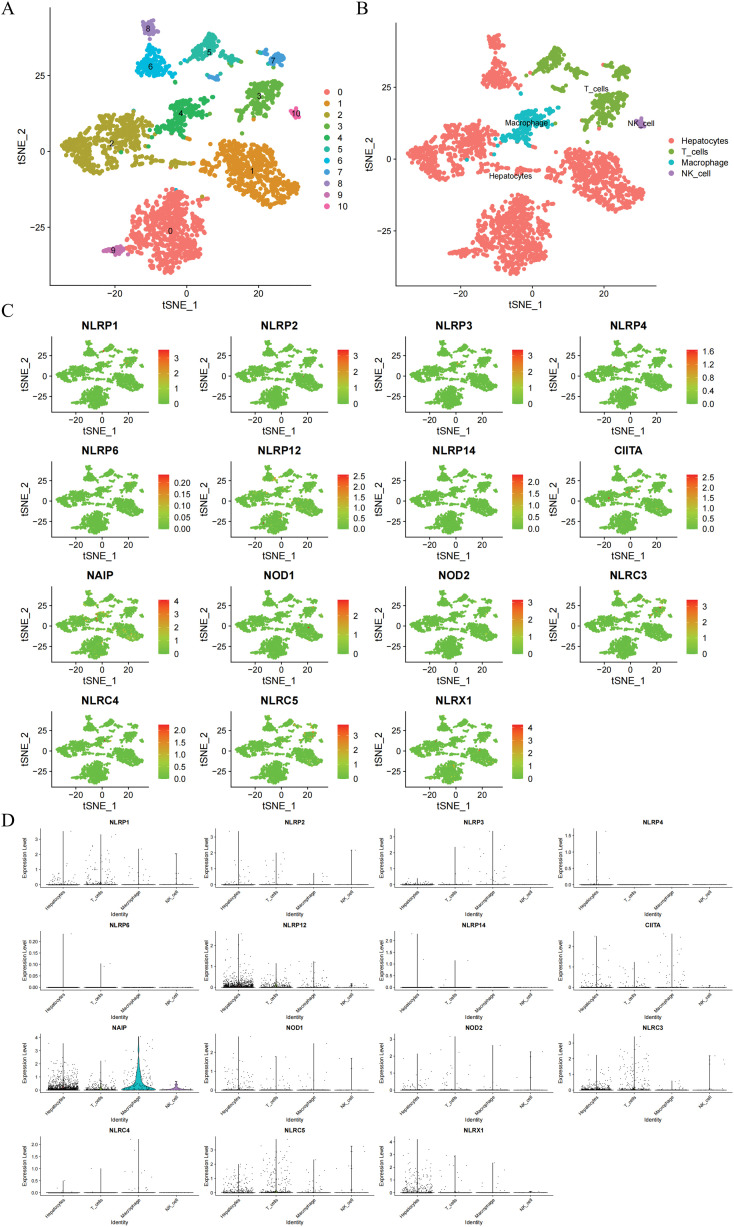
Transcriptional distribution of NLR family genes in HCC single-cell populations. (**A**) t-SNE plot displaying 10 distinct cell clusters identified from HCC single-cell RNA-seq data. (**B**) Annotation of the 10 clusters into four major cell types—hepatocytes, macrophages, natural killer (NK) cells, and T cells—based on the ‘SingleR’ package. (**C**) Feature plots showing the expression of 18 NLR family genes across different cell clusters. (**D**) Violin plots displaying the expression distribution of each NLR family gene across the four annotated cell types

At first, the study utilized t-SNE to conduct dimension reduction and clustering on the scRNA-seq data, which led to the formation of 10 clusters (Fig. 2A). Subsequently, we exploited the ‘SingleR’ package to automatically classify these 10 clusters into four cell types, including hepatocytes, macrophages, natural killer cells, and T cells (Fig. 2B). Based on the results, the study explored the expression distribution of NLR family members in these cell types and employed bubble plots to display the varying expression levels of NLR family genes across different cell types (Fig. 2C,D). In summary, NLRP4 is primarily expressed in hepatocytes, while NLRP6 and NLRP14 are exclusively found in hepatocytes and T cells. The remaining NLR family genes exhibit varying degrees of expression in various cell types.

### 3.3 GO Analysis and KEGG Analysis of Differentially Expressed Genes (DEGs) between Different Subtypes

**Figure 3:**
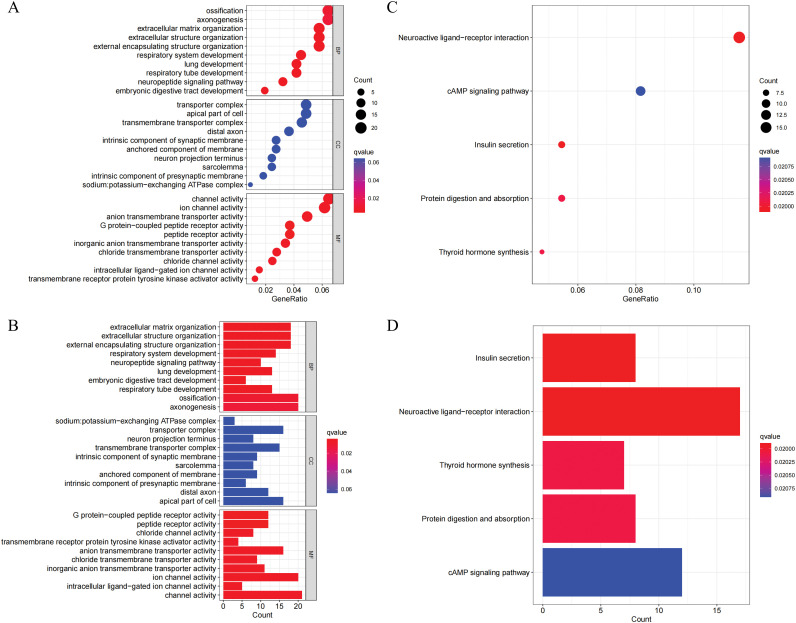
GO and KEGG enrichment analysis of differentially expressed genes (DEGs) between NLR subtypes. (**A–B**) GO enrichment analysis results presented as a bubble plot (**A**) and a bar plot (**B**), showing the top enriched terms in three GO categories: biological processes (BP), cellular components (CC), and molecular functions (MF). (**C–D**) KEGG pathway enrichment analysis of DEGs, shown as a bubble plot (**C**) and a bar plot (**D**). Significantly enriched pathways include neuroactive ligand-receptor interaction, cAMP signaling pathway, and insulin secretion

It was discovered that there were 360 genes that showed differential expression between the C1 subtype and the C2 subtype through differential analysis. This was determined by applying the filter criteria of |Log_2_(Foldchange)| > 2 and *p* < 0.001. Subsequently, an analysis of GO and KEGG was performed on these DEGs. The analysis of GO results indicated that the DEGs were mainly enriched in biological processes related to extracellular matrix organization, extracellular structure organization, and external encapsulating structure organization (Fig. 3A,B). The cellular components were predominantly localized in the sodium:potassium-exchanging ATPase complex, the transporter complex, and the neuron projection terminus. The MFs were primarily associated with G protein-coupled peptide receptor activity, peptide receptor activity, and chloride channel activity. According to the KEGG analysis, the DEGs were significantly enriched in neuroactive ligand-receptor interaction, the cAMP signaling pathway, and insulin secretion (Fig. 3C,D).

### 3.4 NLR Score Based on DEGs and Comprehensive Analysis Involving Tumor Microenvironment

**Figure 4:**
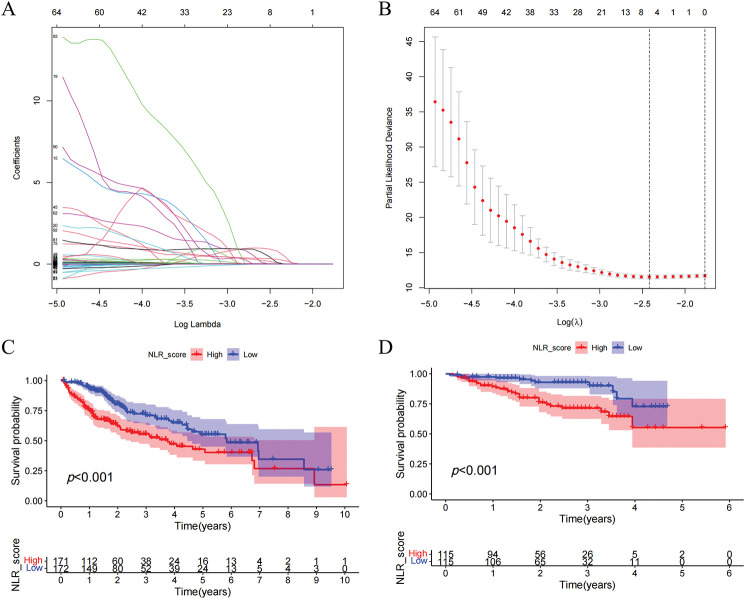

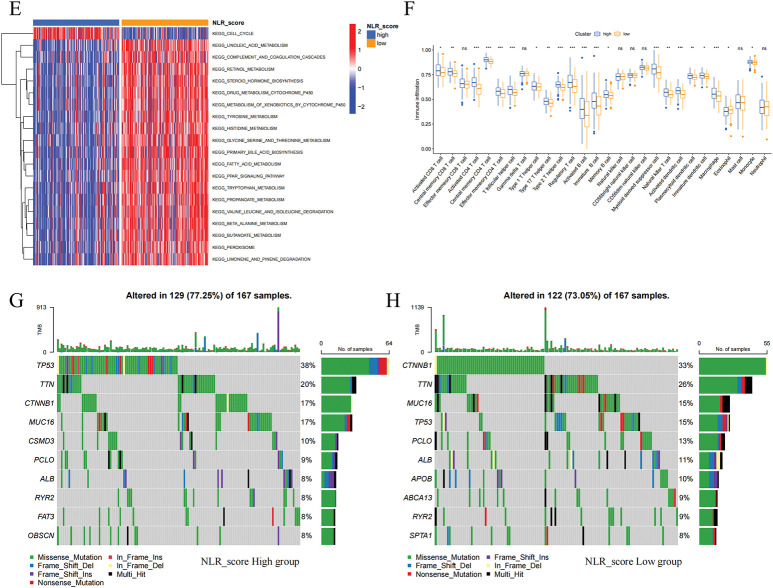
Construction and validation of the NLR_score and its association with prognosis, immune infiltration, and mutation landscape in HCC. (**A**, **B**) LASSO Cox regression analysis was applied to identify hub prognostic DEGs among 123 DEGs. (**A**) The LASSO coefficient profiles of candidate genes. (**B**) 10-fold cross-validation for selecting the optimal λ value. (**C**, **D**) Kaplan–Meier survival curves comparing overall survival (OS) between high and low NLR_score groups in the TCGA-HCC cohort (**C**) and the ICGC-HCC cohort (**D**). (**E**) Heatmap showing GSVA enrichment results of hallmark pathways between NLR_score groups. The low-score group is enriched in cell cycle–related pathways, while the high-score group is enriched in lipid metabolism and inflammatory pathways. (**F**) Boxplot displaying ssGSEA-based infiltration levels of 28 immune cell types in high vs. low NLR_score groups. (**G**, **H**) Oncoplots showing the top 10 most frequently mutated genes in the high NLR_score group (**G**) and low NLR_score group (**H**), respectively. ns represents no significant (*p* > 0.05), *represents *p* < 0.05; **represents *p* < 0.01; ***represents *p* < 0.001

Prognostic analysis was conducted on the DEGs. The prognosis of HCC patients was influenced by 123 DEGs according to the results of the univariate Cox regression analysis. On this basis, we additionally employed LASSO regression analysis and multivariate Cox regression analysis to screen and ultimately identified 5 hub DEGs (Fig. 4A,B). According to multivariate Cox regression analysis, the construction formula of the NLR_score was as follows: HAVCR1 expression × 0.051, ZNF488 expression × 1.571, TEX15 expression × 0.621, G6PD expression × 0.011, and FGF9 expression × 2.327. Subsequently, we evaluated the ability of the NLR_score to predict the prognosis of patients diagnosed with HCC. Based on the median NLR_score value, the patients with HCC were categorized into two groups: the high NLR_score group and the low NLR_score group. In the TCGA-HCC cohort, it was noted that patients belonging to the high NLR_score group demonstrated a considerably reduced overall survival in comparison to individuals in the low NLR_score group (Fig. 4C). The validation cohort ICGC-HCC supported this finding, demonstrating that the NLR_score is a reliable and persistent prognostic marker (Fig. 4D). Furthermore, our analysis utilizing GSVA revealed distinct enrichment patterns in various NLR_score groups. In particular, the group with a low NLR_score exhibited significant enrichment in cell cycle-related pathways, whereas the group with a high NLR_score demonstrated predominant enrichment in linoleic acid metabolism, complement and coagulation cascades, and retinol metabolism (Fig. 4E). Moreover, the single-sample gene set enrichment analysis (ssGSEA) analysis revealed unique distribution patterns of 21 immune cell types across the various groups. Except for neutrophils, the other 20 types of immune cells showed increased levels of infiltration in the high NLR_score group (Fig. 4F). Additionally, we have identified the top 10 genes prone to mutation in both high NLR_score and low NLR_score groups (Fig. 4G,H). Notably, TP53 demonstrated a greater frequency of mutations in the high NLR_score category, whereas CTNNB1 exhibited a higher mutation rate in the low NLR_score group.

### 3.5 Drug Sensitivity Analysis

**Figure 5:**
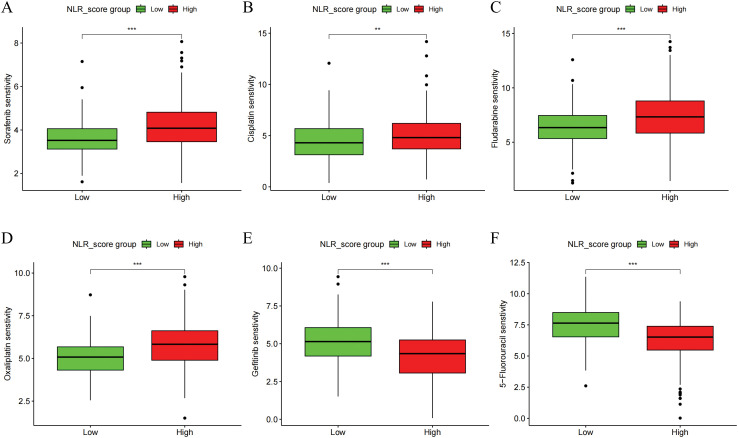
Drug sensitivity analysis of NLR_score subgroups in HCC. Boxplots showing the estimated IC_50_ values of six commonly used chemotherapeutic agents in high and low NLR_score groups. Drug sensitivity analysis was performed using the “pRRophetic” algorithm based on gene expression data. (**A**) Sorafenib (**B**) Cisplatin (**C**) Fludarabine (**D**) Oxaliplatin (**E**) Gemcitabine (**F**) 5-Fluorouracil. Statistical significance was determined using the Wilcoxon rank-sum test. **represents *p* < 0.01; ***represents *p* < 0.001

The IC_50_ values of six frequently utilized chemotherapy drugs in liver cancer patients were assessed across different NLR_score groups. The findings revealed that, when compared to the high NLR_score group, the low NLR_score group displayed decreased IC50 values for sorafenib, cisplatin, fludarabine, and oxaliplatin (Fig. 5A–D), while higher IC50 values were noted for gefitinib and 5-fluorouracil (Fig. 5E,F). These results suggest that HCC patients with low NLR_scores display greater sensitivity to sorafenib, cisplatin, fludarabine, and oxaliplatin, whereas those with high NLR_scores exhibit heightened sensitivity to gefitinib and 5-fluorouracil.

### 3.6 Screening of Key NLR Family Members in HCC and Discovering the Oncogenic Function of NLRP5

**Figure 6:**
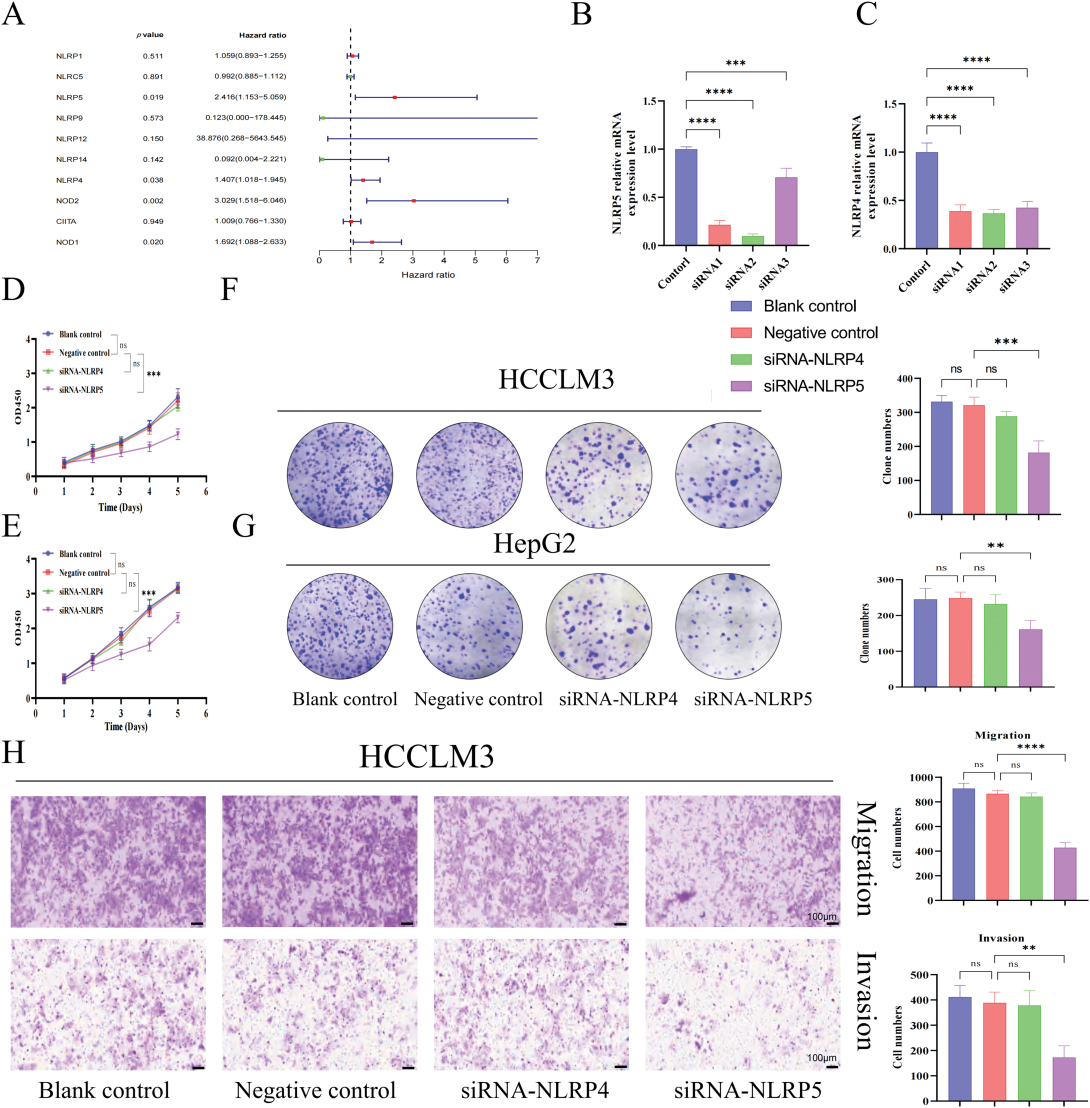

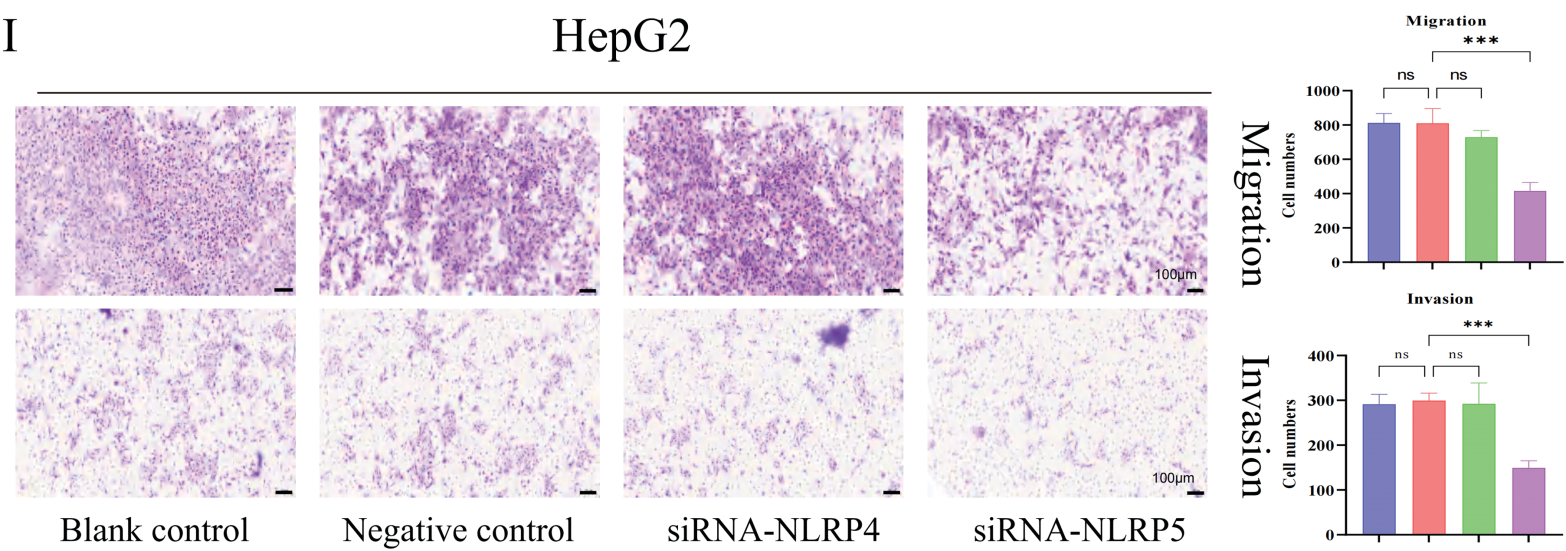
NLRP5 promotes the proliferation, migration, and invasion of hepatocellular carcinoma cells. (**A**) Univariate Cox regression analysis of differentially expressed NLR family genes in HCC. (**B**, **C**) Validation of siRNA-mediated knockdown efficiency of NLRP4 and NLRP5 in HCCLM3 and HepG2 cells using qRT-PCR. siRNA1/2/3 represent three independent siRNA sequences. (**D**, **E**) CCK8 assays were conducted to assess the proliferation of HCCLM3 (**D**) and HepG2 (**E**) cells after transfection with siRNA targeting NLRP4 or NLRP5. (**F**, **G**) Colony formation assays and quantification of colony numbers in HCCLM3 (**F**) and HepG2 **(G**) cells. (**H**, **I**) Transwell migration and Matrigel-coated invasion assays were performed to evaluate the migratory and invasive abilities of HCCLM3 (**H**) and HepG2 (**I**) cells after siRNA transfection. Representative images and statistical analyses are shown. All data are presented as Mean ± SEM. ns represents no significant (*p* > 0.05), **represents *p* < 0.01, ***represents *p* < 0.001, ****represents *p* < 0.0001. All experiments were independently repeated three times

In order to investigate the prognostic importance of NLR family genes in liver cancer, we performed univariate Cox regression analysis on the differentially expressed NLR family genes in HCC (Fig. 6A). The analysis uncovered that NOD1, NOD2, NLRP4, and NLRP5 are risk factors that impact the prognosis. Consequently, our research focused on these four NLR family genes that are associated with prognosis. It is worth noting that NOD1 and NOD2 have been previously studied in the context of HCC [27,28]. Therefore, we aimed to examine the roles of NLRP4 and NLRP5 in HCC. To achieve this, we utilized siRNA transfection to knock down NLRP4 and NLRP5 in the liver cancer cell lines HCCLM3 and HepG2, and subsequently confirmed the knockdown efficiency through RT-qPCR analysis (Fig. 6B,C). CCK8 and colony formation assays were employed to examine the impact of NLRP4 and NLRP5 on the ability of proliferation in HCC cell lines. The findings from the CCK8 assay indicated that diminished expression of NLRP5 notably hindered the proliferation ability of HCCLM3 and HEPG2 cells, whereas reduced expression of NLRP4 did not exert any influence on the proliferation ability of HCC cell lines (Fig. 6D,E). Similarly, the results of the colony formation experiments were comparable (Fig. 6F,G). Subsequently, the transwell assay was employed to investigate the impact of NLRP4 and NLRP5 on the migratory ability. The results from the transwell experiment indicated that the migratory ability of HCCLM3 and HepG2 cells was significantly impaired by a reduction in NLRP5 expression, whereas a decrease in NLRP4 expression had no impact on the ability of HCC cell lines to migrate (Fig. 6H,I). Furthermore, we conducted additional investigations to examine the impact of NLRP4 and NLRP5 on the metastatic potential of HCC cell lines (Fig. 6H,I). The findings revealed that the downregulation of NLRP5 significantly compromised the metastatic capacity of HCCLM3 and HEPG2 cells, while the downregulation of NLRP4 did not exert any discernible influence on the metastatic ability of HCC cell lines. In summary, our experimental results indicate that the proliferation, migration, and invasion abilities of HCC cell lines are controlled by NLRP5, not NLRP4.

### 3.7 Potential Biological Role of NLRP5 in HCC

**Figure 7:**
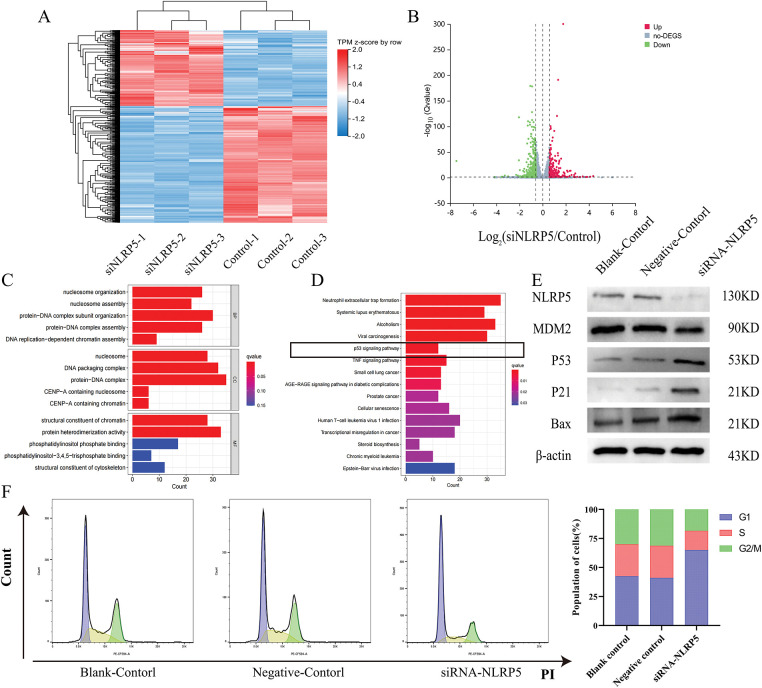

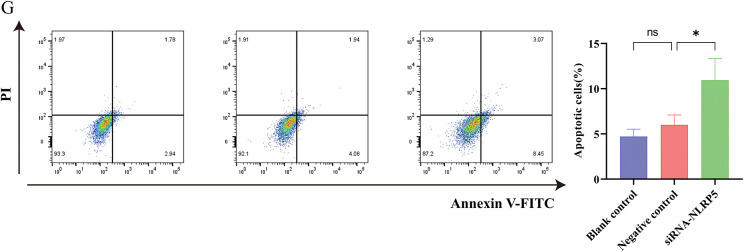
NLRP5 regulates cell cycle progression and apoptosis through the p53 signaling pathway. (**A**) Heatmap showing the differentially expressed genes (DEGs) between the siNLRP5 and control groups based on transcriptome sequencing. (**B**) Volcano plot illustrating significantly upregulated (**red**) and downregulated (**green**) genes in the siNLRP5 group compared to the control group. (**C**) Gene Ontology (GO) enrichment analysis of DEGs in terms of biological processes (BP), cellular components (CC), and molecular functions (MF). (**D**) KEGG pathway enrichment analysis of DEGs showing significant enrichment in the p53 signaling pathway and other cancer-related pathways. (**E**) Western blot analysis of NLRP5, MDM2, P53, P21, and Bax expression levels in HCC cells HCCLM3 following NLRP5 knockdown. (**F**) Cell cycle analysis was performed using flow cytometry, and the distribution of cells in G1, S, and G2/M phases. (**G**) Apoptosis analysis by Annexin V-FITC/PI double staining between the siNLRP5 group and the control group. All data are presented as Mean ± SEM. ns represents no significant (*p* > 0.05), *represents *p* < 0.05. All experiments were independently repeated three times

For the purpose of gaining a deeper understanding of the biological function of NLRP5, we employed transcriptome sequencing to identify DEGs influenced by the knockdown of NLRP5. The findings revealed that the expression levels of 306 genes exhibited an increase, while the expression levels of 470 genes exhibited a decrease (Fig. 7A,B). For the DEGs, we conducted GO and KEGG analyses to further investigate the functions. The GO analysis results revealed that the primary BPs associated with the DEGs included protein-DNA complex subunit organization, nucleosome organization, protein-DNA complex assembly, nucleosome assembly, and DNA replication-dependent chromatin assembly. The main CCs were identified as protein-DNA complex and DNA packaging complex, nucleosome, CENP-A-containing nucleosome, and CENP-A-containing chromatin. Additionally, the MFs were found to be protein heterodimerization activity, structural constituent of chromatin, phosphatidylinositol phosphate binding, structural constituent of cytoskeleton and phosphatidylinositol−3, 4, 5−trisphosphate binding (Fig. 7C). According to the KEGG analysis, the DEGs were found to be prominently enriched in multiple signaling pathways implicated in cancer initiation and progression, including neutrophil extracellular trap formation and the p53 signaling pathway (Fig. 7D). Notably, the p53 signaling pathway attracted particular attention. Consequently, additional experiments were conducted to validate these findings, and Western blot analysis demonstrated that downregulating NLRP5 expression led to a reduction in the ubiquitination of MDM2 to P53, leading to a notable rise in the levels of P53 and its associated downstream proteins P21 and Bax (Fig. 7E). Subsequently, Flow cytometry was applied to perform functional verification of the cell cycle (Fig. 7F). After the implementation of siRNA to diminish NLRP5 expression, the cells transfected with NLRP5-siRNA exhibited a noteworthy increase in G1 phase, in contrast to both the blank control group and the negative control group. This arrest was accompanied by a reduction in the proportion of cells in the S phase and G2/M phase, thereby elucidating the attenuated proliferative capacity. Furthermore, the Annexin-V/PI double staining assay was employed to assess the apoptosis of cells transfected with NLRP5-siRNA (Fig. 7G). Our findings suggest a significant increase in apoptosis among the cells that were transfected with NLRP5-siRNA. In conclusion, these results indicated that NLRP5 plays a role in modulating cell cycle and apoptosis through regulating the p53 pathway, thereby contributing to the progression of malignant biological processes in HCC.

### 3.8 Reducing NLRP5 to Suppress Liver Cancer Growth In Vivo

**Figure 8:**
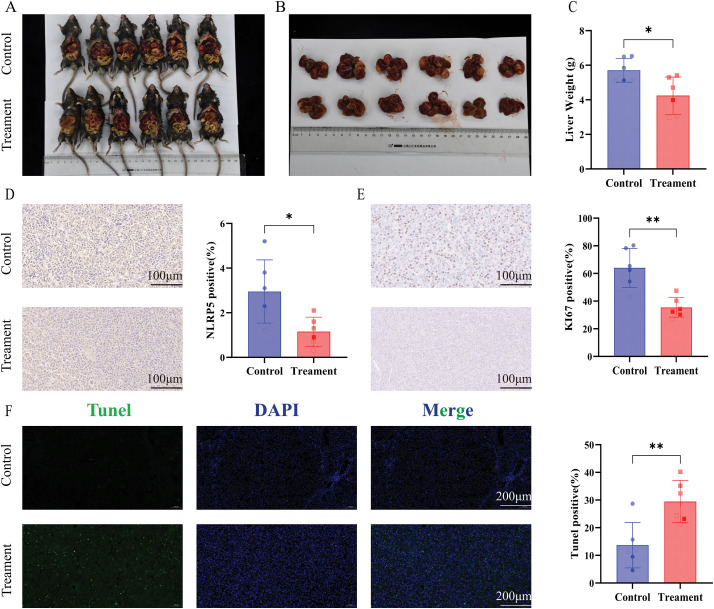
NLRP5 knockdown suppresses tumor growth and proliferation while promoting apoptosis *in vivo*. (**A**, **B**) Representative images of tumor-bearing Alb-Cre^+^/MYC^+^ mice in the control and siNLRP5 treatment groups (**A**), and excised liver tumors (**B**). (**C**) Quantification of liver weight of the siNLRP5-treated group and the control group. (**D**) Immunohistochemical staining for NLRP5 in liver tumor tissues revealed. Quantification of NLRP5-positive cells is shown in the right panel. (**E**) Immunohistochemical staining of Ki67, a proliferation marker. Quantification of Ki67-positive cells is shown in the right panel. (**F**) Representative TUNEL assay fluorescence images and quantification are shown. All data are presented as Mean ± SEM. ns represents no significant (*p* > 0.05), *represents *p* < 0.05, **represents *p* < 0.01

To further investigate the *in vivo* function of NLRP5, we acquired mice with spontaneous liver cancer expressing Alb-Cre^+^/MYC^+^, as detailed in the Methods section. The newborn mice were randomly divided into the siNLRP5 treatment group and the negative control treatment group. After receiving a 3-week course of injection therapy, the mice were euthanized. The experimental findings revealed a significant reduction in tumor volume upon administration of siNLRP5 (Fig. 8A–C). The expression level of NLRP5 in the liver was assessed using immunohistochemistry, and results demonstrated a significant decrease in NLRP5 expression in the liver following siNLRP5 treatment (Fig. 8D). Additionally, the siNLRP5 treatment group exhibited a notable reduction in Ki67 expression, a widely employed marker for assessing tumor proliferation (Fig. 8E). Furthermore, the level of apoptosis in liver cancer cells was evaluated through TUNEL staining. The experimental findings demonstrated a significant rise in the proportion of liver cancer tissue necrosis following siNLRP5 treatment, in contrast to the control group (Fig. 8F). Consequently, it can be concluded that NLRP5 holds promise as a therapeutic target *in vivo*.

## 4 Discussion

HCC remains a global health challenge characterized by its high incidence and mortality rates. Despite significant advancements, the intricate processes governing its initiation and progression are still not fully elucidated. The NLR family has garnered considerable attention for its multifaceted roles in cancer development, presenting promising avenues for molecular therapeutic targeting [29]. While the NLRP3 inflammasome, the most extensively investigated member of the NLR family, has been closely linked to HCC progression [30,31], and various gene set-based investigations have aimed to predict HCC prognosis and the immune microenvironment for clinical decision-making [32,33]. A robust prognostic classification and model specifically derived from the NLR family has been lacking. To bridge this gap, the study developed a powerful prognostic tool for HCC. This study first used NMF to classify HCC patients into two distinct subtypes (C1 and C2) based on differentially expressed NLR family genes. This classification effectively stratified patient prognosis, with the C1 subtype showing significantly shorter overall survival than C2. Building on this, we developed a robust NLR_score from the DEGs between these subtypes. This score comprises HAVCR1, ZNF488, TEX15, G6PD, and FGF9. Most of these genes have established roles in HCC; for instance, HAVCR1 and ZNF488 are known prognostic markers [34,35], G6PD promotes HCC growth by suppressing ferroptosis [36], and FGF9 drives proliferation and metastasis [37]. While TEX15 is a novel inclusion, the established roles of other genes bolster the accuracy of our NLR_score.

The study then rigorously validated the NLR_score’s prognostic power across TCGA-HCC and ICGC-HCC cohorts, consistently finding that a high NLR_score correlated with significantly reduced OS. Delving into the biological distinctions via Gene Set Variation Analysis, we found crucial metabolic and proliferative differences. The high NLR_score group was markedly enriched in linoleic acid metabolism. This pathway is often hijacked by cancer, producing pro-inflammatory lipid mediators and reactive oxygen species that fuel tumorigenesis, proliferation, and foster a pro-tumorigenic inflammatory microenvironment in HCC [38]. This metabolic reprogramming likely underlies the more aggressive phenotype and poorer prognosis in this group. Conversely, the low NLR_score group showed predominant enrichment in cell cycle pathways, potentially indicating a more regulated proliferative state or distinct oncogenic drivers. Our NLR_score also effectively differentiated the tumor immune microenvironment, with a high score associating with specific immune cell profiles that may indicate immune evasion or immunosuppression, thus impacting prognosis and suggesting its potential for guiding immune-related therapies. Furthermore, we identified a significantly higher incidence of TP53 mutations in the high NLR_score group. As a critical tumor suppressor [39], TP53 mutation in HCC is a frequent hallmark of aggressive tumors and worse outcomes [40]. Its elevated prevalence in the high NLR_score group thus substantially validates the score’s prognostic accuracy. Finally, our assessment of drug sensitivity revealed that sorafenib, a first-line HCC therapy, showed superior efficacy in the low NLR_score group, positioning the NLR_score as a potential biomarker for predicting sorafenib response and aiding clinical decision-making.

Our prognostic analysis of individual NLR family genes identified NOD1, NOD2, NLRP4, and NLRP5 as significant risk factors impacting OS in HCC patients. Given the limited research on NLRP4 and particularly NLRP5 in HCC, we conducted a comprehensive investigation into their roles. Our experiments definitively showed that NLRP5, but not NLRP4, significantly promoted the proliferation, migration, and invasion of HCC cell lines. *In vivo*, specific siRNA-mediated suppression of NLRP5 in Myc-driven spontaneous liver cancer mice led to a significant reduction in tumor volume, unequivocally confirming its potent oncogenic function. Mechanistically, we revealed that NLRP5 contributes to HCC progression by regulating the p53 signaling pathway. Specifically, NLRP5 downregulation increased the protein levels of p53 and its downstream targets P21 and Bax, resulting in G1 phase cell cycle arrest and enhanced apoptosis. This uncovers a novel mechanistic insight into NLRP5’s role in HCC. The pro-tumorigenic role of NLRP5 in HCC contrasts sharply with NLRP3, which is generally considered tumor-suppressive through its inflammasome-activating properties [41,42]. This highlights the functional heterogeneity within the NLR family in cancer. While NLRP3 acts via inflammasome pathways, NLRP5 appears to modulate cell proliferation and apoptosis through non-inflammasome-dependent mechanisms. Moreover, despite NLRP5’s well-established critical roles in germline biology, such as oocyte maturation and embryonic development, where it is implicated in maintaining genome integrity [43,44]. Its function in somatic carcinogenesis has been largely undefined. Our study uniquely reveals its pro-oncogenic role in HCC through the modulation of the p53 pathway. Further research is needed to determine whether NLRP5 plays similar oncogenic roles in other malignancies, particularly in reproductive-related tumors such as ovarian tumors and gestational trophoblastic diseases such as hydatidiform mole, where its physiological expression is highly enriched in oocytes and early embryonic cells. Exploring these dimensions could uncover broader implications of NLRP5 as a context-specific oncogene and facilitate the development of precision therapies targeting the NLRP5-p53 signaling axis in diverse tumor types. More importantly, our study will provide a theoretical basis for future clinical decision-making in the treatment of HCC using nanoRNA therapy targeting NLRP5-p53 signaling axis [45].

Despite these compelling findings, our study has certain limitations: First, we did not comprehensively evaluate NLRP5’s impact on tumor-infiltrating immune cells, such as macrophages and CD8^+^ T cells, which are crucial components of the tumor microenvironment. Second, we did not explore the potential synergistic anti-tumor effects of combining NLRP5 inhibition with established HCC therapies like sorafenib. Third, while we provided preliminary evidence that NLRP5 influences the p53 signaling pathway, the specific upstream regulatory factors of NLRP5 and its direct binding partners in this axis remain to be fully elucidated, warranting further in-depth investigation. In summary, our study establishes a robust and stable NLR_score for reliably predicting HCC patient prognosis, effectively differentiating the tumor immune microenvironment, and offering valuable insights for clinical decision-making, including sorafenib sensitivity. Critically, we are the first to systematically reveal NLRP5’s potent oncogenic function in HCC, demonstrating its role through the regulation of the p53 signaling pathway to impact cell cycle control and apoptosis. This work significantly advances our understanding of the intricate roles of NLR family members in cancer, particularly NLRP5. Future research should prioritize rigorous exploration of the NLRP5-p53 axis’s molecular mechanisms and evaluation of its therapeutic potential for precision medicine in HCC.

## Data Availability

The datasets used or analyzed during the current study are available from the corresponding authors upon reasonable request.

## References

[1] Sung H, Ferlay J, Siegel RL, Laversanne M, Soerjomataram I, Jemal A, et al. Global Cancer Statistics 2020: GLOBOCAN estimates of incidence and mortality worldwide for 36 cancers in 185 countries. CA Cancer J Clin. 2021;71(3):209–49. doi:10.3322/caac.21660; 33538338

[2] Yang JD, Hainaut P, Gores GJ, Amadou A, Plymoth A, Roberts LR. A global view of hepatocellular carcinoma: trends, risk, prevention and management. Nat Rev Gastroenterol Hepatol. 2019;16(10):589–604. doi:10.1038/s41575-019-0186-y; 31439937 PMC6813818

[3] Tang M, Hu X, Wang Y, Yao X, Zhang W, Yu C, et al. Ivermectin, a potential anticancer drug derived from an antiparasitic drug. Pharmacol Res. 2021;163(4613):105207. doi:10.1016/j.phrs.2020.105207; 32971268 PMC7505114

[4] Yin Z, Dong C, Jiang K, Xu Z, Li R, Guo K, et al. Heterogeneity of cancer-associated fibroblasts and roles in the progression, prognosis, and therapy of hepatocellular carcinoma. J Hematol Oncol. 2019;12(1):101. doi:10.1186/s13045-019-0782-x; 31547836 PMC6757399

[5] Li Q, Ding C, Cao M, Yang F, Yan X, He S, et al. Global epidemiology of liver cancer 2022: an emphasis on geographic disparities. Chin Med J. 2024;137(19):2334–42. doi:10.1097/cm9.0000000000003264; 39227359 PMC11441870

[6] Chen G, Shaw MH, Kim YG, Nuñez G. NOD-like receptors: role in innate immunity and inflammatory disease. Annu Rev Pathol. 2009;4(1):365–98. doi:10.1146/annurev.pathol.4.110807.092239; 18928408

[7] Gong T, Liu L, Jiang W, Zhou R. DAMP-sensing receptors in sterile inflammation and inflammatory diseases. Nat Rev Immunol. 2020;20(2):95–112. doi:10.1038/s41577-019-0215-7; 31558839

[8] Swanson KV, Deng M, Ting JP. The NLRP3 inflammasome: molecular activation and regulation to therapeutics. Nat Rev Immunol. 2019;19(8):477–89. doi:10.1038/s41577-019-0165-0; 31036962 PMC7807242

[9] Huang Y, Xu W, Zhou R. NLRP3 inflammasome activation and cell death. Cell Mol Immunol. 2021;18(9):2114–27. doi:10.1038/s41423-021-00740-6; 34321623 PMC8429580

[10] He Y, Hara H, Núñez G. Mechanism and regulation of NLRP3 inflammasome activation. Trends Biochem Sci. 2016;41(12):1012–21. doi:10.1016/j.tibs.2016.09.002; 27669650 PMC5123939

[11] Guo H, Callaway JB, Ting JP. Inflammasomes: mechanism of action, role in disease, and therapeutics. Nat Med. 2015;21(7):677–87. doi:10.1038/nm.3893; 26121197 PMC4519035

[12] de Carvalho Ribeiro M, Szabo G. Role of the inflammasome in liver disease. Annu Rev Pathol. 2022;17(1):345–65. doi:10.1146/annurev-pathmechdis-032521-102529; 34752711 PMC10501045

[13] Xiao Y, Zhao C, Tai Y, Li B, Lan T, Lai E, et al. STING mediates hepatocyte pyroptosis in liver fibrosis by Epigenetically activating the NLRP3 inflammasome. Redox Biol. 2023;62:102691. doi:10.1016/j.redox.2023.102691; 37018971 PMC10106968

[14] Wang Q, Ou Y, Hu G, Wen C, Yue S, Chen C, et al. Naringenin attenuates non-alcoholic fatty liver disease by down-regulating the NLRP3/NF-κB pathway in mice. Br J Pharmacol. 2020;177(8):1806–21. doi:10.1111/bph.14938; 31758699 PMC7070172

[15] Frissen M, Liao L, Schneider KM, Djudjaj S, Haybaeck J, Wree A, et al. Bidirectional role of NLRP3 during acute and chronic cholestatic liver injury. Hepatology. 2021;73(5):1836–54. doi:10.1002/hep.31494; 32748971

[16] Zhong W, Rao Z, Rao J, Han G, Wang P, Jiang T, et al. Aging aggravated liver ischemia and reperfusion injury by promoting STING-mediated NLRP3 activation in macrophages. Aging Cell. 2020;19(8):e13186. doi:10.1097/01.tp.0000699180.94561.4c. 32666684 PMC7431827

[17] Shi L, Zhang S, Huang Z, Hu F, Zhang T, Wei M, et al. Baicalin promotes liver regeneration after acetaminophen-induced liver injury by inducing NLRP3 inflammasome activation. Free Radic Biol Med. 2020;160:163–77. doi:10.1016/j.freeradbiomed.2020.05.012; 32682928

[18] Zhao H, Zhang Y, Zhang Y, Chen C, Liu H, Yang Y, et al. The role of NLRP3 inflammasome in hepatocellular carcinoma. Front Pharmacol. 2023;14:1150325. doi:10.3389/fphar.2023.1150325; 37153780 PMC10157400

[19] Gao Y, Church G. Improving molecular cancer class discovery through sparse non-negative matrix factorization. Bioinformatics. 2005;21(21):3970–5. doi:10.1093/bioinformatics/bti653; 16244221

[20] Sun W, Wang J, Wang Z, Xu M, Lin Q, Sun P, et al. Combining WGCNA and machine learning to construct basement membrane-related gene index helps to predict the prognosis and tumor microenvironment of HCC patients and verifies the carcinogenesis of key gene CTSA. Front Immunol. 2023;14:1185916. doi:10.3389/fimmu.2023.1185916; 37287981 PMC10242074

[21] Butler A, Hoffman P, Smibert P, Papalexi E, Satija R. Integrating single-cell transcriptomic data across different conditions, technologies, and species. Nat Biotechnol. 2018;36(5):411–20. doi:10.1038/nbt.4096; 29608179 PMC6700744

[22] Mayakonda A, Lin DC, Assenov Y, Plass C, Koeffler HP. Maftools: efficient and comprehensive analysis of somatic variants in cancer. Genome Res. 2018;28(11):1747–56. doi:10.1101/gr.239244.118; 30341162 PMC6211645

[23] Maeser D, Gruener RF, Huang RS. OncoPredict: an R package for predicting in vivo or cancer patient drug response and biomarkers from cell line screening data. Brief Bioinform. 2021;22(6):bbab260. doi:10.1093/bib/bbab260; 34260682 PMC8574972

[24] Wu T, Li H, Su C, Xu F, Yang G, Sun K, et al. Microbiota-derived short-chain fatty acids promote LAMTOR2-mediated immune responses in macrophages. mSystems. 2020;5(6):10–1128. doi:10.1128/msystems.00587-20; 33144310 PMC7646525

[25] Mei Y, Zhou C, Liang CY, Lu GM, Zeng MS, Wang JJ, et al. A method to establish a c-Myc transgenic mouse model of hepatocellular carcinoma. MethodsX. 2020;7(5):100921. doi:10.1016/j.mex.2020.100921; 32489910 PMC7256637

[26] Moossavi M, Parsamanesh N, Bahrami A, Atkin SL. Sahebkar A. Role of the NLRP3 inflammasome in cancer. Mol Cancer. 2018;17(1):158. doi:10.1186/s12943-018-0900-3; 30447690 PMC6240225

[27] Ma X, Qiu Y, Zhu L, Zhao Y, Lin Y, Ma D, et al. NOD1 inhibits proliferation and enhances response to chemotherapy via suppressing SRC-MAPK pathway in hepatocellular carcinoma. J Mol Med. 2020;98(2):221–32. doi:10.1007/s00109-019-01868-9; 31872284

[28] Zhou Y, Hu L, Tang W, Li D, Ma L, Liu H, et al. Hepatic NOD2 promotes hepatocarcinogenesis via a RIP2-mediated proinflammatory response and a novel nuclear autophagy-mediated DNA damage mechanism. J Hematol Oncol. 2021;14(1):9. doi:10.1186/s13045-020-01028-4; 33413510 PMC7791875

[29] Karki R, Man SM, Kanneganti TD. Inflammasomes and cancer. Cancer Immunol Res. 2017;5(2):94–9. doi:10.1158/2326-6066.cir-16-0269; 28093447 PMC5593081

[30] Tang YL, Tao Y, Zhu L, Shen JL, Cheng H. Role of NLRP3 inflammasome in hepatocellular carcinoma: a double-edged sword. Int Immunopharmacol. 2023;118(9):110107. doi:10.1016/j.intimp.2023.110107; 37028274

[31] Gao Z, Feng SR, Chen JF, Li XG, Shi YH, Tang Z, et al. Inhibition of autophagy in macrophage promotes IL-1β-mediated hepatocellular carcinoma progression via inflammasome accumulation and self-recruitment. Biomed Pharmacother. 2023;161:114560. doi:10.1016/j.biopha.2023.114560; 36940618

[32] He Q, Yang J, Jin Y. Immune infiltration and clinical significance analyses of the coagulation-related genes in hepatocellular carcinoma. Brief Bioinform. 2022;23(4):bbac291. doi:10.1093/bib/bbac291; 35849048

[33] Wan S, Lei Y, Li M, Wu B. A prognostic model for hepatocellular carcinoma patients based on signature ferroptosis-related genes. Hepatol Int. 2022;16(1):112–24. doi:10.21203/rs.3.rs-614871/v1. 34449009

[34] Yang L, Zeng LF, Hong GQ, Luo Q, Lai X. Construction of a novel clinical stage-related gene signature for predicting outcome and immune response in hepatocellular carcinoma. J Immunol Res. 2022;2022:6535009. doi:10.1155/2022/6535009; 35865652 PMC9296277

[35] Wang T, Dai L, Shen S, Yang Y, Yang M, Yang X, et al. Comprehensive molecular analyses of a macrophage-related gene signature with regard to prognosis, immune features, and biomarkers for immunotherapy in hepatocellular carcinoma based on WGCNA and the LASSO algorithm. Front Immunol. 2022;13:843408. doi:10.3389/fimmu.2022.843408; 35693827 PMC9186446

[36] Cao F, Luo A, Yang C. G6PD inhibits ferroptosis in hepatocellular carcinoma by targeting cytochrome P450 oxidoreductase. Cell Signal. 2021;87:110098. doi:10.1016/j.cellsig.2021.110098; 34325001

[37] Paur J, Valler M, Sienel R, Taxauer K, Holzmann K, Marian B, et al. Interaction of FGF9 with FGFR3-IIIb/IIIc, a putative driver of growth and aggressive behaviour of hepatocellular carcinoma. Liver Int. 2020;40(9):2279–90. doi:10.1111/liv.14505; 32378800 PMC7496895

[38] Nava Lauson CB, Tiberti S, Corsetto PA, Conte F, Tyagi P, Machwirth M, et al. Linoleic acid potentiates CD8^+^ T cell metabolic fitness and antitumor immunity. Cell Metab. 2023;35(4):633–50.e639. doi:10.1016/j.cmet.2023.02.013; 36898381

[39] Long J, Wang A, Bai Y, Lin J, Yang X, Wang D, et al. Development and validation of a TP53-associated immune prognostic model for hepatocellular carcinoma. EBioMedicine. 2019;42(2):363–74. doi:10.1016/j.ebiom.2019.03.022; 30885723 PMC6491941

[40] Sen N, Satija YK, Das S. p53 and metabolism: old player in a new game. Transcription. 2012;3(3):119–23. doi:10.4161/trns.20094; 22771946 PMC3616081

[41] Wang H, Ma L, Su W, Liu Y, Xie N, Liu J. NLRP3 inflammasome in health and disease (Review). Int J Mol Med. 2025;55(3):48. doi:10.3892/ijmm.2025.5489; 39930811 PMC11781521

[42] Xu Z, Kombe Kombe AJ, Deng S, Zhang H, Wu S, Ruan J, et al. NLRP inflammasomes in health and disease. Mol Biomed. 2024;5(1):14. doi:10.1186/s43556-024-00179-x; 38644450 PMC11033252

[43] Docherty LE, Rezwan FI, Poole RL, Turner CL, Kivuva E, Maher ER, et al. Mutations in NLRP5 are associated with reproductive wastage and multilocus imprinting disorders in humans. Nat Commun. 2015;6(1):8086. doi:10.1038/ncomms9086; 26323243 PMC4568303

[44] Mu J, Wang W, Chen B, Wu L, Li B, Mao X, et al. Mutations in NLRP2 and NLRP5 cause female infertility characterised by early embryonic arrest. J Med Genet. 2019;56(7):471–80. doi:10.1136/jmedgenet-2018-105936; 30877238

[45] Yuan Y, Sun W, Xie J, Zhang Z, Luo J, Han X, et al. RNA nanotherapeutics for hepatocellular carcinoma treatment. Theranostics. 2025;15(3):965–92. doi:10.7150/thno.102964; 39776807 PMC11700867

